# Seaweed extract and arbuscular mycorrhiza co-application affect the growth responses and essential oil composition of *Foeniculum vulgare* L.

**DOI:** 10.1038/s41598-023-39194-3

**Published:** 2023-07-24

**Authors:** Farzad Rasouli, Yousef Nasiri, Mohammad Bagher Hassanpouraghdam, Mohammad Asadi, Taher Qaderi, Amini Trifa, Maciej Strzemski, Sławomir Dresler, Małgorzata Szczepanek

**Affiliations:** 1 Department of Horticulture, Faculty of Agriculture, University of Maragheh, Maragheh, 5518183111 Iran; 2 Department of Plant Production and Genetics, Faculty of Agriculture, University of Maragheh, Maragheh, 5518183111 Iran; 3 Department of Analytical Chemistry, Medical University of Lublin, 20-093 Lublin, Poland; 4 Department of Plant Physiology and Biophysics, Institute of Biological Sciences, Faculty of Biology and Biotechnology, Maria Curie-Sklodowska University, 20-033 Lublin, Poland; 5 Department of Agronomy, Bydgoszcz University of Science and Technology, 85-796 Bydgoszcz, Poland

**Keywords:** Plant sciences, Plant physiology, Plant symbiosis

## Abstract

The influence of arbuscular mycorrhiza fungi (AMF) inoculation, seaweed extract (SWE) foliar use, and their co-applications were evaluated on the growth-associated traits, antioxidant potential, essential oil profile, and the nutrients content of fennel plants. A factorial experiment was conducted as a completely randomized design with two factors and four replications in the greenhouse. The factors were: AMF inoculation (not inoculated and inoculated with 5 g kg^−1^) and SWE foliar application (0, 0.5, 1.5, or 3 g L^−1^). The highest root colonization percentage was recorded in plants treated with AMF + 3 g L^−1^ of SWE. The top recorded plant height, leaf number, leaf dry weight, biomass, thousand seed weight (TSW), total soluble proteins and total soluble carbohydrates content, antioxidant activity, and essential oil content belonged to AMF + 3 g L^−1^ of SWE. Furthermore, the co-application of AMF + SWE resulted in a considerable enhancement of the photosynthetic pigments content and, in N, P, K, Fe, Zn, and Mn contents in the shoots and roots. The GC-FID and GC–MS analysis revealed that (*E*)-anethole (73.28–76.18%), fenchone (5.94–8.26%), limonene (4.64–6.58%), methyl chavicol (2.91–3.18%), and (*Z*)-*β*-ocimene (1.36–2.01%) were the principal essential oil constituents. The top (*E*)-anethole and fenchone contents were obtained by AMF + SWE. Altogether, the simultaneous application of AMF and SWE could be introduced as an environment-friendly strategy to reach reliable growth responses, especially in fennel plants' enriched with some precious essential oil constituents.

## Introduction

Safety, quality, and efficacy have recently been recognized as remarkable criteria for the use of medicinal and aromatic plants in industrialized and developing countries^1^. Standardization and the safety evaluation of constituents derived from medicinal plants can help the emergence of a new era of health domain for the treatment of various diseases in the future^2^. The therapeutic potential of these plants depends on the secondary metabolites, which are divided into three main groups: phenolics, alkaloids, and terpenoids, which act as antioxidants and also have protective attributes against several ailments. They are effective against various diseases^3^. In recent years, medicinal plant cultivation has become more important due to the role of those plants in modern and traditional medicine. *Apiaceae* is one of the important families of plants and, due to their aromatic compounds, they are extensively utilized in various pharmaceutical, food, health, and flavoring industries^4^. Fennel (*Foeniculum vulgare* Mill.) is one of the utmost valued medicinal plants belonging to the *Apiaceae* (*Umbelliferaceae*) family that is predominantly in use for its seeds and essential oils in the numerous pharmaceutical, food, and cosmetic industries. This plant is native to the Mediterranean, Europe, and Egypt and is planted all over the world^5^. Fennel seeds contain proteins, lipids, carbohydrates, fiber, minerals (Ca, K, Na, Fe, P), and vitamins such as vitamin C, vitamin E, vitamin B6, thiamin, riboflavin, and niacin^6^. Fennel preparations have many therapeutic properties including liver protection, anti-tumor, anti-stress, anti-diabetes, and anti-aging^7^. Its most important constituents are (*E*)-anethole, fenchone, limonene, terpinene, and α-phellandrene^8^. Nowadays, due to the significance of the mass production of medicinal plants and their use as natural substances in human health, it is essential to employ various methods of planting and nutrition regimes which enhance the essential oil biosynthesis and their valuable components in medicinal plants^9^. The global approach has changed toward the establishment of a sustainable agricultural system, especially with the development of novel agricultural management methods. The use of growth biostimulants is a novel procedure to provide essential nutrients for plants^10^. Microbial biostimulants and their by-products affect the crops' growth, yield, quality, and nutrient use efficiency^11^.

Seaweed extract (SWE) contains some of the macro- and micro-nutrients, which possibly play an effective role in the growth and development of plants^12^. Moreover, SWE contains stimulatory compounds such as auxins, cytokinins, betaines, and gibberellins, which influence the plant’s growth and productivity^13^. Also, there are many reports related to the different effects of SWE on plants. In an experiment conducted by Mafakheri^14^ on the fenugreek plant, it was reported that using SWE increased the percentage and yield of essential oil (EO). In another study, in parsley (*Petroselinum crispum* (Mill.) Fuss); chlorophyll content, seed yield, EO percentage, and yield were significantly affected by SWE foliar spray^15^. Also, the results of Mostafa^16^ showed a significant increase in the yield, yield components, and EO content (EOC) of fennel (*F. vulgare*) due to the foliar application of SWE compared to the control plants. The idea is that the reason for improving the performance of plants with biological stimuli can be related to the improvements in the biochemical processes in the plants and soil, the activation of some growth-stimulating enzymes, the transfer of ions, and as a result, the promotion of photosynthesis potential^17^. Ansary et al.^18^ reported that foliar spraying of basil and mint with SWE increased their vegetative growth and EOC.

Plants evolved a wide range of mechanisms to overcome adverse environmental conditions, one of the most significant is their association with symbiotic microorganisms. Mycorrhizal fungi interact with the roots which drastically improves water absorption and nutrent acquisition and also, protects the plants against root pathogens^19^. They biotrophically colonize plant roots and, by creating an external mycelium, help plants to more effectively absorb mineral elements and water. On the other hand, the fungus receives its photosynthetic materials from the plant, and this relationship considerably affects the crops' endurance and growth, particularly in nutrient-deficient soils, thus increasing crop productivity in degraded agricultural systems^20^. In fact, due to its phosphatase activity and organic compounds that dissolve insoluble phosphate, this fungus can improve the effective surface of the root and the ability to absorb phosphorus by the plant and create a symbiotic association with the roots of 90% of plants^21^. Arbuscular mycorrhizal fungi (AMF) act a fundamental role in complex networks of biological interactions that link plant and soil communities and represent key biological agents in natural and agricultural ecosystems^22^. AMF, as an obligate biotroph, depends on host plants to complete their life cycle and obtain organic compounds, mainly sugars, and lipids from the plants^23^. AMF improves the physical, chemical, and biological quality of the soil and plays a significant role in providing humus, N, and P to poor soils and finally making the non-absorbable P available to the plant^24^. Root inoculation of plants with AMF impacts the content and quality of important active compounds such as lipids, phenolics, terpenoids, or alkaloids derived from plants^25^. These are related to improved nutrition, the balance of phytohormones, the induction of specific metabolic pathways, and some morphological changes^26^. The role of AMF in improving the growth and yield of aromatic and medicinal plants is largely understood. Many studies have been conducted on the effect of these fungi on the yield and EO of plants^27^. In this case, during a study conducted on green tea, it was found that arbuscular mycorrhiza significantly increased the concentration of EO and biomass of the plant^28^. Also, in another study, it was observed that inoculation of fennel plants with AMF significantly enhanced seed yield and plant biomass^29^. In the peppermint plant, AMF treatment improved the root colonization, growth, essential oil yield, and nutrient acquisition of the plant^30^. In addition, the arbuscular mycorrhizal fungus increases the efficiency of photosynthesis in the host plant, and it has been reported that the quantitative and qualitative characteristics of the lettuce plants were significantly affected by the SWE and AMF co-treatments^31,32^. Also, in coriander, in addition to the positive effects on biomass performance and root colonization; AMF significantly increased the alpha-pinene content compared to control plants^33^.

Since there is a lack of information on the co-application of AMF and SWE on the morphological and physiological responses and EO constituents of fennel; this experiment aimed to look into the influence of *Funneliformis mosseae* inoculation and *Ascophyllum nodosum* foliar spraying on growth parameters, photosynthetic pigments content, total antioxidant activity, macro-, and micro-elements content, and EO composition of fennel.

## Results

### Arbuscular Mycorrhizal Fungi (AMF) colonization

**Figure 1:**
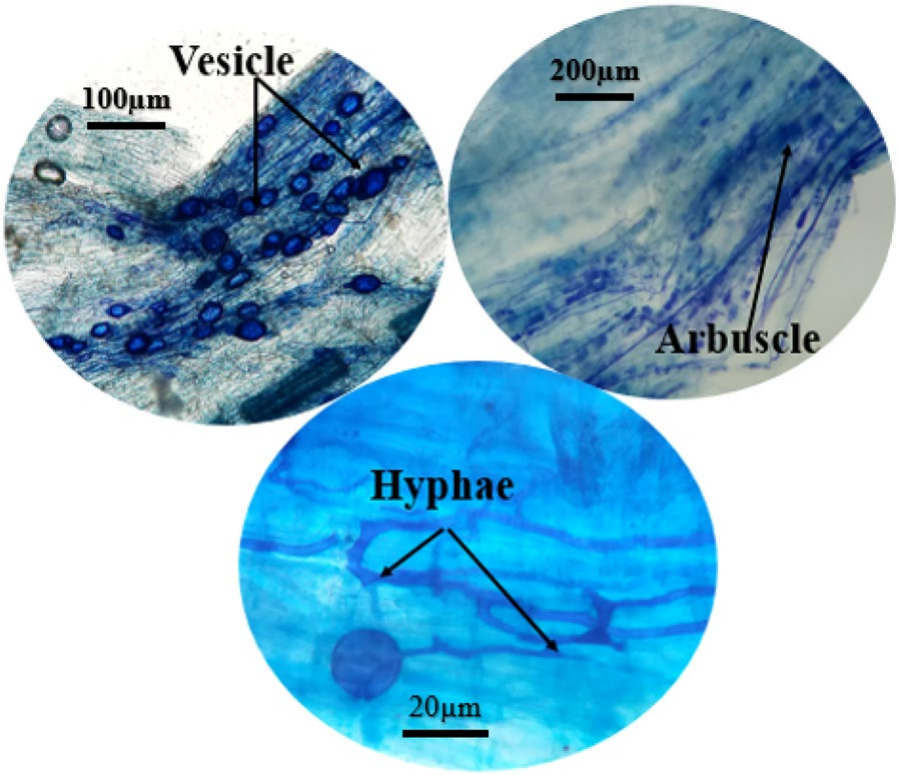
Microscopic images of the dyed fennel root fragments for uncovering arbuscular mycorrhizae (*F. mosseae*) colonization.

Microscopic images of fennel root parts were traced to reveal arbuscular mycorrhizal colonization, which is shown in Fig. 1. Photographs documented the AMF structures and hyphae.

### Growth characteristics

**Figure 2:**
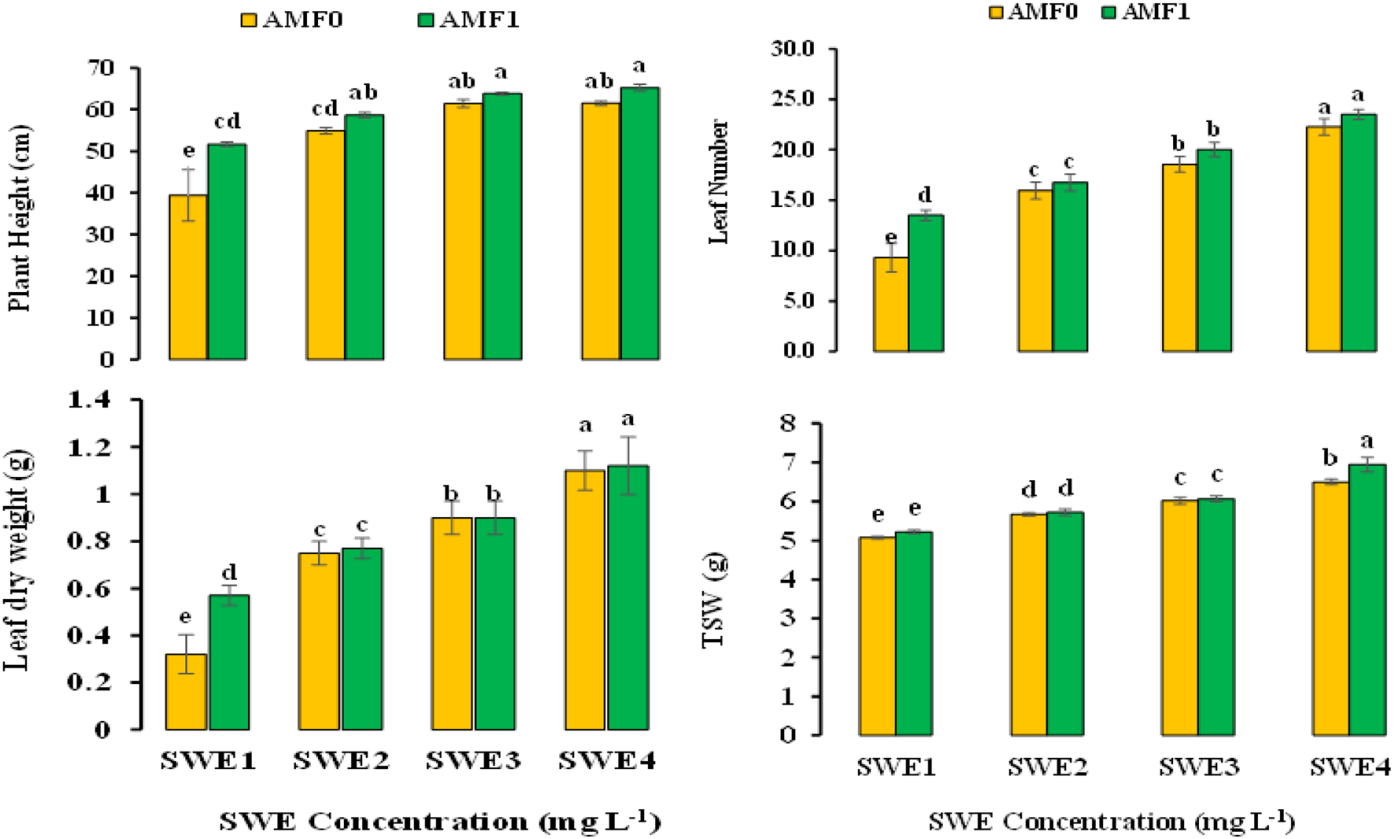
The influence of arbuscular mycorrhiza fungus (AMF) + seaweed extract (SWE) co-application on plant height, leaf number, leaf dry weight, and thousand seed weight (TSW) of fennel. Dissimilar letters reveal significant differences according to the LSD test at p < 0.05. AMF 0 and AMF 1 assign to those with no mycorrhiza and with mycorrhiza inoculation, and SWE1, SWE2, SWE3, and SWE4 refer to 0, 0.5, 1.5, and 3 mg L^−1^ of SWE treatment.

Based on the results, the application of AMF and SWE significantly influenced the growth traits, i.e., plant height, leaf number, leaf dry weight, biological yield (BY), and thousand seed weight (TSW) of fennel (Table 1). Moreover, the co-application of SWE and AMF improved plant height, leaf number, leaf dry weight, and TSW by 65, 152, 250, 77, and 37% over the control plants, respectively. The highest data for the growth-related traits were obtained in the fennel plants treated with 3 mg L^−1^ SWE + AMF inoculation. There was no considerable difference in the leaf number and leaf dry weight of plants treated with 3 mg L^−1^ SWE, with and without AMF application (Fig. 2).

**Table 1:** The influence of seaweed extract (SWE) foliar spraying on the growth attributes of fennel plants under arbuscular mycorrhiza (AMF) inoculation.

S.O.V	df	Plant height	Leaf number	Leaf dry weight	TSW
AMF	1	580.06**	188.19**	0.61**	3.48**
SWE	3	201.80**	29.64**	0.03*	0.24**
AMF × SWE	3	47.99**	4.74**	0.03*	0.07**
Error	24	6.854	0.94	0.008	0.01
CV %		4.57	5.57	10.74	1.75

*, ** and ns, significant at the *p* ≤ 5% and 1% and non-significant, respectively. S.O.V. and df refer to the source of variation and degree of freedom, respectively.

### Photosynthesis pigments content

The results showed that the content of chlorophyll a, b, a + b, and carotenoids content was significantly influenced by SWE and AMF. Foliar application of SWE + AMF inoculation improved photosynthetic pigments content compared to plants sprayed with distilled water and without AMF. The highest of Chl a, b, a + b, and carotenoids content was observed in the plants exposed to SWE foliar application (3 mg L^-1^) + AMF, which increased by 121, 145, 129 and 343% compared to the control plants, respectively (Table 2).

**Table 2:** The influence of seaweed extract (SWE) foliar spraying on photosynthetic pigments content of fennel plants under arbuscular mycorrhiza (AMF) inoculation.

SWE (mg L^−1^)	AMF	Chl *a*(mg g^−1^ FW)	Chl *b* (mg g^−1^ FW)	Chl *a* + *b* (mg g^−1^ FW)	Carotenoids (mg g^−1^ FW)
0	0	0.502 ± 0.087f.	0.310 ± 0.067f.	0.812 ± 0.151g	0.078 ± 0.017e
	1	0.707 ± 0.010e	0.468 ± 0.015e	1.175 ± 0.016ef	0.196 ± 0.014d
0.5	0	0.754 ± 0.001de	0.515 ± 0.013de	1.268 ± 0.013cd	0.201 ± 0.002d
	1	0.812 ± 0.018cd	0.554 ± 0.009cd	1.366 ± 0.022d	0.258 ± 0.020bc
1.5	0	0.873 ± 0.011c	0.594 ± 0.014fbc	1.467 ± 0.021f.	0.246 ± 0.014c
	1	0.958 ± 0.012b	0.632 ± 0.006b	1.590 ± 0.012b	0.283 ± 0.037b
3	0	1.045 ± 0.029a	0.709 ± 0.008a	1.755 ± 0.036c	0.285 ± 0.012b
	1	1.112 ± 0.009a	0.760 ± 0.026a	1.871 ± 0.035a	0.344 ± 0.013a
LSD at 0.05%		2.76	2.12	4.67	1.44
S.O.V					
AMF		**	**	**	**
SWE		**	**	**	**
AMF × SWE		**	**	**	*
CV%		8.71	5.56	8.27	7.86

*, ** indicate significance at *P* ≤ 5% and ≤ 1%, respectively. Different letters in each column indicate a significant difference at *p* ≤ 0.05. SWE, S.O.V., and C.V. refer to seaweed extract, source of variance, and coefficient of variation. AMF 0 and AMF 1 assign to those with no arbuscular mycorrhiza and with arbuscular mycorrhiza inoculation at 5 g kg^−1^ soil.

### Total soluble proteins content

The findings revealed that the total soluble proteins content was significantly influenced by the simultaneous application of SWE and AMF. The total soluble proteins content increased (up to 60%) in the fennel plant subjected to 3 mg L^−1^ of SWE + AMF inoculation (Table 3).

**Table 3:** The influence of seaweed extract (SWE) foliar spraying on proteins and carbohydrates content, antioxidant activity, and essential oil (EO) content of fennel plants under arbuscular mycorrhiza (AMF) inoculation.

SWE (mg L^-1^)	AMF	Total soluble proteins content (mg g^−1^ FW)	Total soluble Carbohydrates content (mg g^−1^ FW)	Total antioxidant enzymes activity (%)	Essential oil content (EOC) (%)
0	0	6.72 ± 0.07f.	13.22 ± 0.64e	32.46 ± 0.15g	1.15 ± 0.10f.
	1	7.52 ± 0.04e	18.08 ± 0.26d	46.98 ± 0.10ef	1.71 ± 0.01e
0.5	0	8.39 ± 0.02d	19.72 ± 0.13d	50.72 ± 0.06cd	1.83 ± 0.01de
	1	8.77 ± 0.03c	21.87 ± 0.33c	54.65 ± 0.08d	1.89 ± 0.06d
1.5	0	9.51 ± 0.02b	23.15 ± 0.209fc	58.68 ± 0.02f.	1.99 ± 0.03cd
	1	9.71 ± 0.01b	25.16 ± 0.25b	63.58 ± 0.03b	2.05 ± 0.05c
3	0	10.50 ± 0.03a	26.13 ± 0.16b	70.18 ± 0.08c	2.25 ± 0.021b
	1	10.77 ± 0.11a	35.61 ± 0.86a	74.84 ± 0.03a	2.47 ± 0.03a
LSD at 0.05%		0.328	1.68	4.67	0.159
S.O.V					
AMF		**	**	**	**
SWE		**	**	**	**
AMF × SWE		*	**	**	*
CV %		6.72	4.38	7.87	4.83

** indicate significance at *P* ≤ 1%. Different letters in each column indicate a significant difference at *p* ≤ 0.05. SWE, S.O.V., and C.V. refer to seaweed extract, source of variance, and coefficient of variation. AMF 0 and AMF 1 assign to those with no arbuscular mycorrhiza and with arbuscular mycorrhiza inoculation at 5 g kg^−1^ of soil.

### Total soluble carbohydrates content

The trait was significantly affected by SWE + AMF employment. The maximum total soluble carbohydrates content was recorded in the plants supplemented with 3 mg L^-1^ of SWE + AMF (increased up to 169.5% compared to the control) (Table 3).

### Total antioxidant activity

The total antioxidant activity was significantly increased due to the co-application of SWE + AMF. Under the SWE foliar spraying (3 mg L^−1^) + inoculation of AMF, the total antioxidant activity was enhanced (up to 130.5% compared to the control plants) (Table 3).

### Essential oil content

The interactions of SWE + AMF significantly improved the EO content in the fennel seeds. The EO content was enhanced by 114.7% with the co-application of SWE + AMF compared to the control plants (Table 3).

### Macro- and micro-nutrients content

The results showed that macro- and micro-nutrients content was significantly influenced by the foliar application of SWE and AMF inoculation. The N and P content of the shoot and roots as well as K content of the shoots were increased by SWE + AMF. So, the highest recorded data for these traits were recorded at 3 mg L^−1^ SWE + AMF application (Table 4). While, the lowest N and P content of the shoot and root were observed in the control plants. SWE at 3 mg L^−1^ with and without AMF attained the highest K content of the root. The co-application of SWE and AMF increased the shoot N, P, and K content by 274%, 331%, and 239%, respectively. Moreover, the co-treatments enhanced the root N, P, and K content by 278, 130, and 275%, respectively. The findings showed that the highest Fe and Zn content of the shoot and root were recorded in the foliar application of SWE (3 mg L^−1^) with and without AMF inoculation and also, in SWE at 1.5 mg L^−1^ + AMF (Table 5). While, the lowest data for those traits were observed in the control fennel plants, and the plant inoculated with AMF without SWE foliar application. Finally, the shoots' and roots' Mn content was enhanced by 239 and 175% compared to the control plants, respectively.

**Table 4:** The influence of seaweed extract (SWE) foliar spraying on macro-nutrients content (mg g^−1^ DW) of fennel plants under arbuscular mycorrhizal (AMF) inoculation.

SWE (mg L^-1^)	AMF	N		P		K	
		Shoot	Root	Shoot	Root	Shoot	Root
0	0	0.897 ± 0.09f.	1.01 ± 0.8d	0.029 ± 0.04g	1.22 ± 0.06f.	12.0 ± 0.09f.	20.5 ± 0.05g
	1	1.50 ± 0.04de	1.61 ± 0.05c	0.050 ± 0.02e	1.32 ± 0.01d	19.5 ± 0.01e	24.7 ± 0.04f.
0.5	0	1.33 ± 0.03e	1.44 ± 0.01c	0.063 ± 0.06d	1.57 ± 0.03c	23.0 ± 0.03d	29.5 ± 0.01e
	1	1.80 ± 0.01cd	1.92 ± 0.03bc	0.066 ± 0.04b	1.89 ± 0.05b	27.5 ± 0.05c	35.2 ± 0.03d
1.5	0	1.82 ± 0.02cd	1.93 ± 0.05bc	0.077 ± 0.07f.	2.08 ± 0.02e	33.7 ± 0.01bc	41.0 ± 0.07c
	1	2.18 ± 0.5bc	2.30 ± 0.7b	0.093 ± 0.09e	2.35 ± 0.01d	35.7 ± 0.04b	52.5 ± 0.06b
3	0	2.21 ± 0.04b	2.45 ± 0.04b	0.114 ± 0.01c	2.56 ± 0.04c	33.5 ± 0.02b	54.0 ± 0.02ab
	1	3.36 ± 0.07a	3.82 ± 0.06a	0.125 ± 0.02a	2.81 ± 0.07a	40.7 ± 0.06a	56.5 ± 0.04a
LSD at 0.05%		0.388	0.551	0.053	0.092	3.19	3.21
S.O.V							
AMF		**	**	**	**	**	**
SWE		**	**	**	**	**	**
AMF × SWE		*	*	*	**	*	**
CV%		12.21	10.83	8.44	5.12	7.13	6.15

*, ** indicate significance at *P* ≤ 5% and ≤ 1%, respectively. Different letters in each column indicate a significant difference at *p* ≤ 0.05. SWE, S.O.V., and CV assign to seaweed extract, source of variance, and coefficient of variation. AMF 0 and AMF 1 assign to those with no arbuscular mycorrhiza and with arbuscular mycorrhiza inoculation at 5 g kg^−1^ of soil.

**Table 5:** The influence of seaweed extract (SWE) foliar spraying on micro-nutrients content (mg g^−1^ DW) in fennel plants under arbuscular mycorrhiza (AMF) inoculation.

SWE (mg L^−1^)	AMF	Fe		Zn		Mn	
		Shoot	Root	Shoot	Root	Shoot	Root
0	0	0.042 ± 0.04d	0.116 ± 0.08e	0.024 ± 0.02d	0.105 ± 0.03e	0.015 ± 0.5g	0.013 ± 0.3g
	1	0.095 ± 0.02c	0.184 ± 0.03d	0.053 ± 0.04cd	0.131 ± 0.05d	0.028 ± 0.4e	0.022 ± 0.2ef
0.5	0	0.126 ± 0.03bc	0.219 ± 0.05cd	0.040 ± 0.06cd	0.121 ± 0.04cd	0.024 ± 0.05f.	0.023 ± 0.05ef
	1	0.138 ± 0.05bc	0.233 ± 0.06bd	0.092 ± 0.05ac	0.136 ± 0.08 cd	0.033 ± 0.07e	0.027 ± 0.06e
1.5	0	0.138 ± 0.01bc	0.248 ± 0.05bc	0.066 ± 0.03bd	0.136 ± 0.01c	0.036 ± 0.05d	0.029 ± 0.03cd
	1	0.169 ± 0.04ab	0.275 ± 0.02ab	0.093 ± 0.01ac	0.149 ± 0.03ac	0.040 ± 0.06b	0.035 ± 0.05b
3	0	0.192 ± 0.01a	0.276 ± 0.01ab	0.109 ± 0.02ab	0.158 ± 0.02ab	0.037 ± 0.05c	0.034 ± 0.01c
	1	0.218 ± 0.06a	0.312 ± 0.06a	0.125 ± 0.05a	0.167 ± 0.04a	0.045 ± 0.08a	0.047 ± 0.06a
LSD at 0.05%		0.053	0.206	0.172	0.297	1.47	2.24
S.O.V							
AMF		**	**	**	^ns^	**	**
SWE		**	**	**	**	**	**
AMF × SWE		**	*	*	**	**	*
CV%		6.21	10.18	11.29	7.14	5.04	5.36

ns, * and ** indicated no significant difference, significant at *P* ≤ 5% and *P* ≤ 1%, respectively. Different letters in each column indicate a significant difference at *p* ≤ 0.05. SWE, S.O.V., and C.V. assign to seaweed extract, source of variance, and coefficient of variation. AMF 0 and AMF 1 assign to those with no arbuscular mycorrhiza and with arbuscular mycorrhiza inoculation at 5 g kg^−1^ of soil.

### Essential oil (EO) composition

Based on the GC–MS and GC-FID analyses, a total of 18 components were identified in the EO of fennel, accounting for 92.6–98.3% of the total compositions (Table 6). The results revealed that (E)-anethole (73.28–76.18%) was the main ingredient of fennel EO. In addition, fenchone (5.94–8.26%), limonene (4.64–6.58%), methyl chavicol (2.91–3.18%), and (Z)-β-ocimene (1.36–2.01%) were identified as other predominant constituents of fennel EO. The highest (E)-anethole and fenchone contents were obtained with foliar spraying of SWE (3 g L^−1^) and AMF inoculation. The least content for the mentioned constituent was identified with control plants. The highest limonene content (6.58%) belonged to the foliar SWE treatment (3 g L^−1^) + AMF inoculation. In contrast, the lowest limonene (4.64%) content was recorded for control plants (Table 6).

**Table 6:** The influence of arbuscular mycorrhiza fungi (AMF 0 and 1; without and with AMF) inoculation and seaweed extract (SWE; 0, 0.5, 1.5 and 3 mg L^−1^) foliar spraying on EO compounds of fennel plants.

No	Components	RT (min)	RI	RI literature	SWE_0_ + AMF_0_	SWE_0_ + AMF_1_	SWE_0.5_ + AMF_0_	SWE_0.5_ + AMF_1_	SWE_1.5_ + AMF_0_	SWE_1.5_ + AMF_1_	SWE_3_ + AMF_0_	SWE_3_ + AMF_1_
1	*a*-Thujene	17.84	924	923	0.31 ± 0.04	0.52 ± 0.14	0.14 ± 0.16	0.25 ± 0.26	0.23 ± 0.04	0.10 ± 0.16	0.39 ± 0.13	0.43 ± 0.06
2	Camphene	18.38	946	948	0.72 ± 0.03	0.48 ± 0.11	0.59 ± 0.13	0.45 ± 0.01	0.60 ± 0.17	0.66 ± 0.09	0.71 ± 0.17	0.24 ± 0.03
3	Sabinene	18.73	969	970	0.49 ± 0.07	0.56 ± 0.09	0.52 ± 0.03	0.42 ± 0.03	0.46 ± 0.05	0.52 ± 0.03	0.59 ± 0.03	0.28 ± 0.01
4	*β*-Pinene	13.12	974	976	0.13 ± 0.01	0.12 ± 0.08	0.58 ± 0.01	0.09 ± 0.05	0.49 ± 0.13	0.69 ± 0.08	0.69 ± 0.06	0.10 ± 0.07
5	Myrcene	19.19	988	989	0.40 ± 0.03	0.12 ± 0.03	0.08 ± 0.04	0.07 ± 0.02	0.10 ± 0.02	0.16 ± 0.01	0.11 ± 0.01	0.09 ± 0.01
6	*a*-Phellandrene	19.63	1002	1004	0.06 ± 0.04	0.19 ± 0.13	0.12 ± 0.01	0.11 ± 0.13	0.06 ± 0.03	0.13 ± 0.03	0.12 ± 0.05	0.12 ± 0.10
7	*p*-Cymene	19.78	1022	1019	0.22 ± 0.02	0.20 ± 0.05	0.26 ± 0.12	0.31 ± 0.08	0.25 ± 0.08	0.30 ± 0.12	0.34 ± 0.03	0.47 ± 0.08
8	Limonene	**22.14**	**1024**	**1020**	**5.20 ± 1.48**	**4.95 ± 0.35**	**5.20 ± 0.56**	**4.64 ± 0.45**	**5.39 ± 0.56**	**5.51 ± 0.21**	**5.61 ± 0.54**	**6.58 ± 0.23**
9	1,8-Cineole	22.37	1026	1024	0.09 ± 0.06	0.16 ± 0.04	0.07 ± 0.06	0.08 ± 0.02	0.10 ± 0.03	0.06 ± 0.01	0.05 ± 0.01	0.12 ± 0.05
10	(Z)-*β*-Ocimene	**23.59**	**1032**	**1030**	**1.41 ± 0.23**	**2.01 ± 0.47**	**1.43 ± 0.34**	**1.44 ± 0.58**	**1.36 ± 0.52**	**1.46 ± 0.34**	**1.44 ± 0.29**	**1.49 ± 0.21**
11	ɣ-Terpinene	25.04	1054	1051	0.05 ± 0.03	0.08 ± 0.01	0.05 ± 0.01	0.05 ± 0.01	0.07 ± 0.01	0.05 ± 0.02	0.06 ± 0.02	0.06 ± 0.07
12	Fenchone	**25.41**	**1083**	**1080**	**5.94 ± 1.11**	**7.67 ± 0.27**	**7.97 ± 1.23**	**6.22 ± 1.31**	**6.98 ± 1.12**	**7.97 ± 1.04**	**8.01 ± 0.62**	**8.08 ± 0.36**
13	Camphor	28.51	1141	1139	0.17 ± 0.05	0.16 ± 0.11	0.20 ± 0.02	0.16 ± 0.03	0.17 ± 0.02	0.19 ± 0.01	0.21 ± 0.07	0.10 ± 0.09
14	Methyl chavicol	30.73	1195	1193	**3.03 ± 0.13**	**3.04 ± 1.03**	**2.93 ± 0.67**	**2.95 ± 0.39**	**3.15 ± 0.62**	**3.02 ± 0.19**	**2.91 ± 0.32**	**3.18 ± 0.53**
15	Fenchyl acetate < endo- >	32.53	1218	1220	0.16 ± 0.04	0.16 ± 0.07	0.13 ± 0.05	0.24 ± 0.06	0.18 ± 0.07	0.08 ± 0.05	0.11 ± 0.08	0.25 ± 0.05
16	*p*-Anisaldehyde	33.88	1247	1245	0.08 ± 0.02	0.12 ± 0.02	0.10 ± 0.03	0.23 ± 0.11	0.16 ± 0.04	0.27 ± 0.07	0.12 ± 0.03	0.23 ± 0.11
17	(*E*)-Anethole	**35.11**	**1282**	**1280**	**74.03 ± 1.55**	**74.54 ± 2.72**	**75.97 ± 1.28**	**78.15 ± 2.05**	**76.36 ± 1.43**	**75.36 ± 1.65**	**73.28 ± 1.38**	**76.18 ± 2.32**
18	Germacrene D	43.69	1484	1486	0.14 ± 0.03	0.13 ± 0.02	0.13 ± 0.03	0.15 ± 0.01	0.12 ± 0.05	0.14 ± 0.02	0.16 ± 0.12	0.30 ± 0.02
	Total identified (%)				92.63	95.21	96.47	96.01	96.23	96.67	94.91	98.3

Significant values are in bold.

### PCA analysis of the essential oil constituents

**Figure 3:**
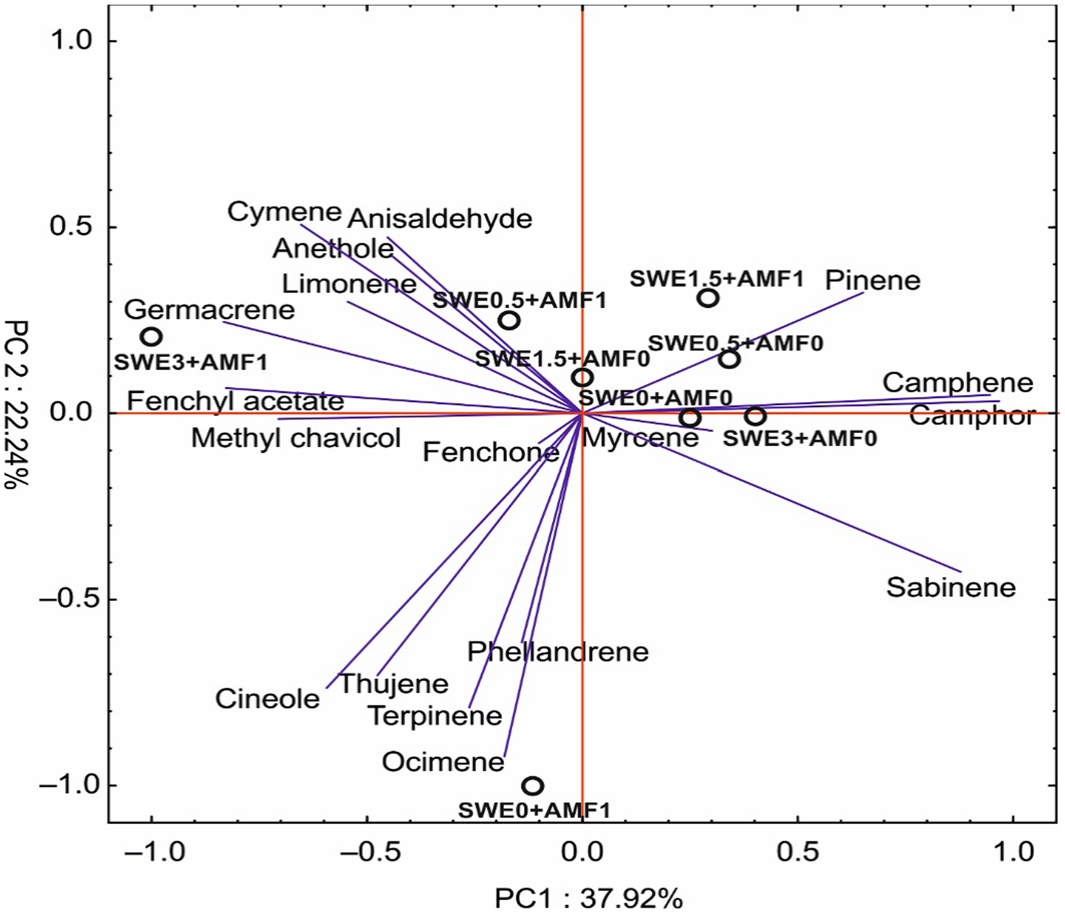
The principal component analysis for fennel essential oil constituents under the co-application of seaweed extract (SWE) and arbuscular mycorrhiza fungus (AMF) inoculation.

The principal component analysis (PCA) of the essential oil composition showed the clear separation of the SWE3 + AMF1 individuals along PC1. The first component explained almost 38% of the total variance, and it was largely loaded by camphene, camphor, pinene, and sabinene (positive correlation) and negatively determined by fenchyl acetate, methyl chavicol, germacrene, limonene, anethole, cymene, and anisaldehyde. In turn, the second component, which explained above 22% of the total variance, facilitated the separation of SWE0 + AMF1 from the other individuals which was characterized by the high content of ocimene, terpinene, thujene, and phellandrene (Fig. 3).

## Discussion

In recent decades, with the development of sustainable agricultural production systems, it’s necessary to use nutrient stimulants or symbiotics to enhance plant productivity and to reach a sustainable ecological environment. The stimulants with their specific physiological and molecular actions improve the absorption and efficiency of nutrients and enhance the yield and quality of crops^34^. The application of a variety of these stimulants, especially microbial and non-microbial stimulants, may effectively improve the growth and productivity of plants. In addition, the effects of the combined application of biostimulants may be antagonistic, additive, or synergistic. These effects have encouraged researchers to design and formulate a variety of biostimulants with specific performances concerning their nutritional and functional qualities^35^.

In this study, the simultaneous utilization of AMF and SWE improved plant height, leaf number, leaf dry weight, BY, and TSW of the fennel plants (Table 1). AMF inoculation enhances the growth and biomass of plants, which could be attributed to an increase in internal hormonal levels^36^. It is believed that growth promotion alters the hormonal status of the plant and thus significantly increases nutrient absorption^37^. In agreement with our results, Golubkina et al.^38^ showed that using AMF boosted growth traits in the hyssop plant. Likewise, Begum et al.^39^ reported that the arbuscular mycorrhiza fungus improved the absorption of water and nutrients in pea plants through a symbiotic relationship and by the creation of an extensive hyphae system. In addition, the improvement of the growth characteristics of the fennel with the application of SWE biostimulant can be assigned to the nutrients and water absorption through the roots and the growth promotion through the action of plant hormones, since, seaweed extract contains micro and macro elements, vitamins, amino acids, and plant hormones such as gibberellin and cytokinin. Also, SWE enhances the division and turgor pressure of meristem cells and hence improves the growth attributes in plants. On the other hand, when sufficient nutrients are available to the plant, the rate of photosynthesis increases, which leads to an enhancement in plant growth and biomass^40,41^. Consistent with our findings, Alam et al.^42^ reported that the foliar utilization of SWE had a considerable impact on the growth of roots and shoots of different plant species, including strawberries, winter canola, and coastal pine. Also, Hussain et al.^13^ reported that growth traits such as root length and stem and root dry weight of tomato plants were increased by using SWE.

The photosynthetic capacity of leaves directly reflects the level of plant productivity^43^. Total chlorophyll and carotenoid content were also increased by the SWE and AMF treatment. These were compared with the findings of Carrasco-Gill et al.,^44^. The use of SWE fertilizer improves the Fe content in the plant tissue, which is one of the essential elements to enhance the chlorophyll content and function in plants^44^. AMF colonization enhances plant photosynthesis potential and thus improves the efficiency of PSII photochemical activity. Similarly, as formerly described, mycorrhizal plants usually have greater photosynthetic pigments content and chlorophyll fluorescence efficiency, and so attain more tolerance to environmental stresses^45^. Foliar application of the SWE could have increased chlorophyll content via inducing absorption of macro and micronutrients, including Mg and Fe necessary for the synthesis of chlorophyll^46^.

Our findings displayed that the co-application of SWE and AMF increased the total protein content in fennel plants. SWE includes several organic ingredients for instance polysaccharides, proteins, and fatty acids which aid in stimulating growth activity (Table 3). Moreover, the protein content of fennel plants was amended with SWE and AMF as a result of increasing nitrate uptake and biological N fixation^47^. SWE and AMF increased total protein content in *Trigonella foenum-graecum* L. ^48^, which agrees with our findings. Also, the improved N uptake and relocation have been repeatedly related to the high protein content^49^. Nonetheless, the increased protein content is related to the enhanced carbohydrate content in plant leaves^50^. The fennel plants treated with SWE and AMF attained a higher carbohydrate content compared to the non-treated ones. The mechanism that has been suggested for AMF concerning plant growth improvement is root mass enhancement that may improve the nutrient absorption and relocation^51^. High total soluble carbohydrates content in the leaves inevitably improves the nitrate assimilation pathway^52^. The enhancement of carbohydrates concentration in AMF-treated plants may be described by the enhanced carbon fixation and the activation of several enzymes^32^. Finally, AMF inoculation improved the net photosynthetic potential as well as the carbohydrate content in *Zenia* plants^53^.

In the present experiment, it is realized that the essential oil content significantly increased due to the co-application of AMF and SWE. AMF improves metabolism and affects the content and quality of secondary metabolites^54^. An indirect mechanism can be influential through the effect on the microbial community and the absorption of the elements in the soil on the content and yield of the EO^55^. On the other hand, the enhanced EO biosynthesis can also be justified by the absorption of the P element, which happened as a result of AMF inoculation^56^. The increase in EO content can also be due to the presence of growth stimulants in the SWE treatment. The nutrients in SWE, such as boron and nitrogen, stimulate the essential oil accumulation in plants^57^. Other nutrients such as phosphorus, and micro-elements like Zn, may induce the growth and enhance the essential oil composition of plants supplemented with SWE^18^. In agreement with our findings, SWE enhanced the EO content and constituent in hyssop plants^58^. Also, Tawfeeq et al.^59^ showed that SWE treatment increased EO compounds (*a*-terpinene and *a*-phellandrene) in rosemary. Similarly, the highest content of EO in Dutch fennel was traced as a result of the co-application of arbuscular mycorrhizal fungus and seaweed extract^60^.

In the current research, N, P, K, Fe, Zn, and Mn contents were improved in the fennel plants supplemented with AMF + SWE. The reason for the increased micronutrients content can be ascribed to the high absorption potential as a result of the symbiosis with the mycorrhizal fungus in addition to the high plant root surface area which also improves the nutrients absorption efficacy. By creating a symbiotic relationship between plants and fungi, AMF improves the uptake of essential elements such as phosphorus and nitrogen by fungal hyphae and simultaneously enhances the nutrients contained in the plant^61^. SWE is also rich in nutrients, such that it has a variety of macro and micro-elements^62^. As a result, the significant enhancement of N, P, K, Fe, Zn, and Mn elements concentration can also be attributed to the additional availability of nutrients. In addition, SWE comprises plant hormones that induce the growth of roots and greatly boost nutrient uptake^63^. Our results are in agreement with the findings of Baslam, et al.^64^ and Abbas et al.^65^. Whether the observed growth stimulation is attributed to the macro- or micro-nutrition available in SWE or to the growth stimulants (i.e. plant hormones) remains to be seen.

## Materials and Methods

### Study site and treatment

The research was run in the greenhouse of Maragheh University, Iran, which is located at 37°23′ north latitude and 46°16′ east longitude, and 1486 m above sea level. The temperature regime was 18–25°C for the night-day, relative humidity was nearby 60–70% and air-conditioned with fans with a 0.5–1.5 m s^-1^. A factorial experiment in the format of a completely randomized design (CRD), with four replications, and three plants per replication was set up. The current research had two factors including the fungus *F. mosseae* inoculation (0 and 5 g kg^−1^ of soil) to the soil at the planting moment and, SWE foliar spraying was applied at 4 levels (0, 0.5, 1.5, and 3 g L^−1^). The AMF was acquired from Zist Fanavar Pishtaz Varian, Karaj, Iran, with one hundred active spores per gram of soil. The SWE (MAX Sailor) was provided by CITYMAX company, Xian, China, which contained organic acid (50%), alginic acid (16%), P (16%), amino acid (16%), N (1%), mannitol (3%), gibberellic acid (300 ppm), auxin (0–20 ppm) and cytokinin (0–30 ppm), and pH was 9–11. The foliar use of SWE was accomplished 30 days after sowing and repeated three times at 7-day intervals. The control fennel plants were grown in the conditions without AMF inoculation and SWE foliar application and they were sprayed with distilled water instead of SWE. The fennel plants were foliar sprayed till the solution or distilled water drops were run off the shoots. 10–20 ml of the SWE treatments on average was foliar sprayed on each plant based on the fennel growth stage. The utilized soil was autoclaved at 121°C under a 1.2 atmosphere for 90 min, to eradicate its microorganisms before sowing the fennel seeds (*F. vulgare* Miller). The fennel seeds were planted in 5 L pots. The fennel plants were watered with tap water every 3–4 days. The soil was sandy clay loam with pH = 8.07, 0.96% organic carbon, 0.08% total N, and 9.06, 485.62, 1.12, 192, and 1.06 mg kg^−1^ of available P, K, Zn, Mn, and Fe, respectively. All methods in the current study were performed by the relevant institutional, national, and international guidelines and legislation.

### Microscopic imaging of root colonization

At first, the root samples of the AMF-inoculated fennels were split into smaller parts (1 cm) and cleaned in a solution of KOH (10%) for 15 min. The specimens were cleansed with tap water and next treated through HCl (2%) at room temperature within 15 min and dyed with trypan blue (0.05%) in 80% lactic acid within 12–14 h. Lastly, the dyed specimens were washed down with water and kept in a mixture comprising water, glycerol, and lactic acid (1:1:1; v:v:v) till the evaluation^66^.

### Measurement of growth parameters and photosynthesis pigments content

The growth characteristics such as plant height, leaf number, leaf dry weight, and thousand seed weight (TSW) were recorded at harvest time. Chlorophyll (Chl) *a*, *b*, *a* + *b,* and carotenoids content were measured via the Arnon method^67^. 0.5 g of fresh plant tissue was grounded with 80% acetone. After centrifugation of the final solution at 10,000 rpm for 10 min, the optical density (OD) was recorded at wavelengths of 470, 645, and 663 nm with a spectrophotometer (UV-1800, Shimadzu, Tokyo, Japan). The photosynthetic pigments content was determined as mg g^-1^ FW with the following equations:

1. Chl *a* = (12.21 A663–2.81 A645) × 2.5 × (v/1000 × w)
2. Chl *b* = (20.13 A645–5.03 A663) × 2.5 × (v/1000 × w)
3. Carotenoids = [(1000A470–3.27 Ca-104Cb)/198] × 2.5 × (v/1000 × w)

### Total soluble proteins content

0.2 g of fresh leaf samples were powdered via liquid nitrogen and squelched in 1.5 ml of sodium phosphate buffer (100 mM and pH = 7.8) along with 1 mM EDTA and 2% (w/v) polyvinyl pyrrolidone. Then, it was centrifuged for 15 min at 4°C and 12,000 rpm, and the supernatant fluid was analyzed to estimate the total soluble proteins content based on the method of Bradford^68^. Finally, the OD was measured at 595 nm with a spectrophotometer and presented as mg g^-1^ FW.

### Total soluble carbohydrates content

0.2 g of fresh fennel leaves sample were crushed using 10 ml of ethanol (95%) for about 1 h in a water bath at a temperature of 80℃ and centrifuged at 12,000 rpm for 10 min. After that, a mixture was made with 1 ml of the supernatant, phenol (0.5%), and 5 ml of sulfuric acid (98%). Finally, the OD was noted at 483 nm via a spectrophotometer. The total soluble carbohydrates content was reported as mg g^−1^ FW^69^.

### Measurement of the total antioxidant activity

Total antioxidant activity was assessed by the FRAP technique^70^. The FRAP reagent contained acetate buffer (300 mM, pH 3.6), 2, 4, 6-tripyridy1-s-triazine (10 mM) dissolved in HCl (40 mM), and ferric chloride hexahydrate (20 mM) in a ratio of 10:1:1 (v:v:v). Then, 3 ml of this reagent was added to 100 µl methanolic extract. After incubation at 37 °C within 15 min, the OD was scanned at 593 nm through a UV–VIS spectrophotometer. Total antioxidant activity was estimated as the percentage of FRAP deactivation.

### Essential oil (EO) extraction

40 g of harvested seeds were extracted over 3 h through the hydro-distillation method described in the British Pharmacopoeia. The oils were dried over anhydrous sodium sulfate to eliminate water droplets and kept at 4 °C in the refrigerator. EO content was determined via equation^71^:

$${\text{EO content }}\left( {w/w\% } \right)\, = \,\left[ {{\text{Extracted EO }}\left( {\text{g}} \right) \, /{4}0{\text{ g of the plant}}} \right]\, \times \,{1}00$$

### Essential oil constituents

Essential oil constituents were analyzed using GC–FID and GC–MS. The GC–MS analysis was done by an Agilent 7990 B gas chromatograph equipped with a 5988A mass spectrometer and an HP-5MS column (0.25 mm i.d., 30 m, 0.25μm f.t., 5% phenyl methylpolysiloxane). The oven temperature programming was: 5 min at 60°C, reaching 240°C at 3°C/min ramp, held for 10 min at the temperature. The helium (carrier gas) flow rate was 1 mL/min; the injector split ratio was 1:30; and the mass range and electron impact (EI) were 40–400 m/z and 70 eV, respectively. The oil constituents were identified by Adams^72^ according to an interactive combination of linear retention indices (RIs), calculated against a homologous series of n-alkanes (C8–C40, Supelco, Bellefonte, CA, USA) and mass spectrum (MS) matching with libraries (ADAMS, WILEY 275 and NIST 17). The GC–FID analysis was carried out by an Agilent 7990 B gas chromatography connected to a flame ionization detector (FID), capillary column VF 5MS (30 m, 0.25 mm i.d., 0.50 μm f.t., 5% phenyl methylpolysiloxane). The above-mentioned oven temperature programming was employed. The injection volume was 1μl of 1:100 (oil: hexane). The quantification of the oil components was carried out by considering the peak area normalization without correction factors^72^.

### Macro- and micro-elements content estimation

A flame photometer was employed to evaluate K content in samples. Also, N and P content were measured through Kjeldahl and the yellow technique, respectively. Vanadate molybdate was applied as an indicator of the yellow method. The amount of P was assessed at a wavelength of 470 nm by a spectrophotometer^73^. Mn, Fe, and Zn contents were determined using an atomic absorption spectrometer (AA-6300 F; Shimadzu, Kyoto, Japan)^74^.

### Statistical analysis

The MSTAT-C ver. 2.1 was employed for the analysis of variance, and the least significant difference test (LSD) at 1% and 5% probability levels were applied for the comparisons of the evaluated traits means. Statistica ver. 13.3. software (TIBCO Software Inc. 2017, Palo Alto, CA, USA) was used to perform principal component analysis (PCA) of the essential oil constituents.

### Ethics approval and consent to participate

All procedures were conducted according the relevant institutional, national, and international guidelines and legislation.

## Conclusions

Overall, the simultaneous application of SWE and AMF enhanced the growth characteristics, protein, carbohydrates, macro- and micro-nutrients contents, and EO composition of the fennel plants. Therefore, the co-treatment of the SWE foliar application + AMF symbiosis can be advised as a promising tool to improve the fennel growth and EO composition. Furthermore, the co-application of AMF and SWE may be considered a reliable biofertilization methodology. All in all, the idea is that the co-application of these biofertilizers, not only reduces the chemical fertilizers' input and their related adverse impression on the ecosystem but also improves the qualitative and quantitative attributes of fennel plants.

## Data Availability

The datasets used and/or analyzed during the current study are available from the corresponding author upon reasonable request.
